# Transcriptome Profiles of *Alternaria oxytropis* Provides Insights into Swainsonine Biosynthesis

**DOI:** 10.1038/s41598-019-42173-2

**Published:** 2019-04-15

**Authors:** Xin Li, Ping Lu

**Affiliations:** College of Life Science and Technology, Inner Mongolia Normal University, Hohhot, 010022 China

## Abstract

Swainsonine (SW) is a toxic alkaloid biosynthesized by the endophytic fungus *Alternaria oxytropis* in *Oxytropis glabra*. The biosynthetic pathway of SW is poorly understood. Saccharopine reductase/dehydrogenase of fungus plays an important role in this pathway. The gene knocked out mutant M1 in *A. oxytropis* was constructed in our previous work. In this study, the transcriptome of wild-strain OW7.8 and M1 was firstly sequenced to understand the biosynthetic pathway and molecular mechanism of SW in *A. oxytropis*. A total of 45,634 Unigenes were annotated. 5 genes were up-regulated and 11,213 genes were down-regulated. 41 Unigenes possibly related to the biosynthesis of SW were identified by data analyzing. The biosynthesis pathway of SW in the fungus was speculated, including two branches of P6C and P2C. Delta1-piperidine-2-carboxylate reductase, lysine 6-dehydrogenase, and saccharopine oxidase/L-pipecolate oxidase were involved in P6C. 1-piperidine-2-carboxylate/1-pyrroline-2- carboxylate reductase [NAD(P)H] and delta1-piperidine-2-carboxylate reductase were involved in P2C. Saccharopine reductase was involved in both. In addition, 1-indolizidineone was considered to be the direct precursor in the synthesis of SW, and the hydroxymethylglutaryl-CoA lyase catalyzed the synthesis of SW. Here we analyzed details of the metabolic pathway of *A. oxytropis* SW, which is of great significance for the follow-up research.

## Introduction

*Oxytropis glabra* DC. is a perennial herb of the genus *Oxytropis*. Many plants contain swainsonine(SW), an indolizidine alkaloid poisonous to livestock. Its cations can bind to mannoside competitively inhibiting the activity of alpha-mannosidase in animal cells^1,2^. Livestock will be poisoned when SW accumulates to certain degree in their body and even dead in serious cases^3,4^. *Oxytropis*, *Astragalus* and other poisonous plants containing SW are collectively called Locowed internationally. The spread of locowed brings huge losses to grassland animal husbandry in the world. It is of great significance to study the toxic mechanism of locowed. In addition, in the medicine and chemical industry, SW has been a tool for studying the synthesis of glycoprotein N-oligosaccharides, and later found to have anti-tumor and immunomodulatory effects, so it has attracted attention^4,5^. SW (C_8_H_15_NO_3_) has a double heterocyclic structure and four chiral configurations. It is easy to form chiral isomers in the process of chemical synthesis and difficult to distinguish them, so it is expensive. Biosynthesis of SW is a new way.

Braun *et al*.^6^ isolated SW-producing endophytic fungus from three locoweed plants of *Oxytropis lambertii*, *Oxytropis sericea* and *Astragalus mollisimus*, and identified them as *Embellisia oxytropis*. Pryor *et al*.^7^ revised them to *Undifilum oxytropis*, and later Woudenberg *et al*.^8^ revised them to *Alternaria oxytropis*. Ping Lu *et al*.^2^ isolated the endophytic fungus from *O*. *glabra* and synthesized SW *in vitro*. The host without this endophytic fungus did not contain SW. Therefore, it was proposed that SW was produced by *A*. *oxytropis* fungus. According to the results of microbiology research and DNA sequence analysis, SW was classified as a group of fungus such as Braun’s endophytic fungus.

In the SW synthesis pathway of *Rhizoctonia Leguminosae* and *Metarhizium anisoplia9*^7–10^, saccharopine reductase/dehydrogenase catalyzes the conversion of lysine to saccharopine. Then it produces alpha-aminohexyldialdehyde, which is one of the precursors of SW synthesis. Therefore, saccharopine reductase is a key enzyme in SW biosynthesis^11^, but its specific metabolic pathway is not clear yet. The saccharopine reductase gene (KJ944635; KY052048) of the endophytic fungus *A*. *oxytropis* was cloned and its deletion vector was constructed by the research group in earlier stage. The deletion mutant M1 was obtained by homologous recombination and protoplast regeneration. With the development of second-generation sequencing technology, OW7.8 and M1 will be used for transcriptome sequencing and analysis in the study to speculate their biosynthetic pathways and screen *A*. *oxytropis* SW synthesis-related genes so as to provide basic data for the study of gene function, which is of great theoretical significance for elucidating the mechanism of SW biosynthesis and important practical value in guiding the control of SW in *Oxytropis glabra* in the future.

## Results

Our previous research found that the saccharopine reductase in *A*. *oxytropis* played an important role in SW biosynthesis. In this study, transcriptome of the wild-type strain OW7.8 and the saccharopine reductase gene disruption mutant M1 were performed. Differential genes related to SW biosynthesis were screened and the SW biosynthesis pathway in the fungus was postulated.

### Fungus transcriptome sequencing and construction of transcriptional information platform

*A*. *oxytropis* transcriptome sequencing was completed on the Illumina HiSeq^TM^4000 high-throughput sequencing platform. Without Raw Reads, 47.91 million Clean Reads in the transcriptome sequencing library were used for assembly of subsequent transcriptome de novo. Blastx comparison (E < 10^−5^) between the assembled 45,637 Unigene sequences and known databases, i.e. Nr, Swiss-Prot, Go, KOG and KEGG were made to obtain the annotation results of Unigene functions (as shown in Table 1).

**Table 1:** Statistics for RNA-Seq based on sequencing, assembly and functional annotation for *A*. *oxytropis*.

Assembling results	Number of unigenes	45,634
	Total length (nt) of total unigenes	57,560,042
	Mean length (nt) of total unigenes	1,261
	N50 (nt) of total unigenes	2,630
Annotation	Unigenes with NR	24,133 (52.88% of 45,634 unigenes)
	Unigenes with NT	24,593 (53.89%)
	Unigenes with KO	9,239 (20.24%)
	Unigenes with Swiss-Prot	22,416 (49.12%)
	Unigenes with KOG	11,866 (26.00%)
	Unigenes with PFAM	21,544 (47.21%)
	Unigenes with GO	21,931 (48.06%)
	Unigenes with KEGG	717 (1.57%)
	Annotated in all Databases	5,486 (12.02% of 45,634 unigenes)

### Sequence analysis

**Figure 1:**
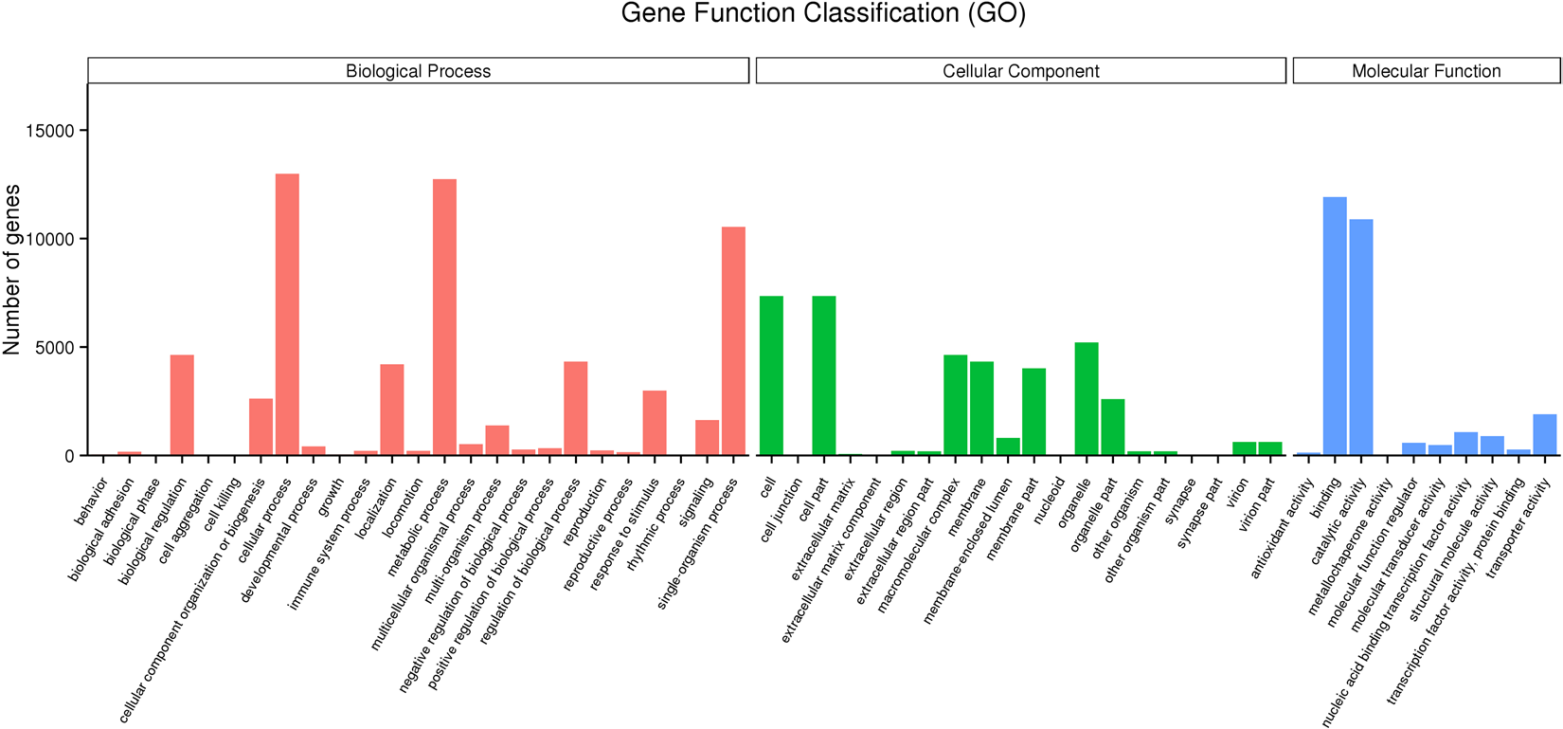
GO categorization of Unigenes. Distribution of genes in different GO function classification.

For comparison of different transcripts, 7,586 DGEs were assigned to 55 GO enrichments, as shown in Fig. 1. Among them, there were 802 DGEs (10.54%) in transmembrane transport biological process. Both transcription factor activity, sequence-specific DNA binding molecular function and nucleic acid binding transcription factor activity molecular function had 500 DEGs. The triglyceride lipase activity molecular-function had the minimum of 27 DGEs, accounting for 0.35%. These metabolic pathways involved the growth and reproduction and the metabolic processes related to the accumulation of secondary metabolites of the endophytic fungus, indicating that these pathways were closely related to the whole development process of *A*. *oxytropis*.

**Figure 2:**
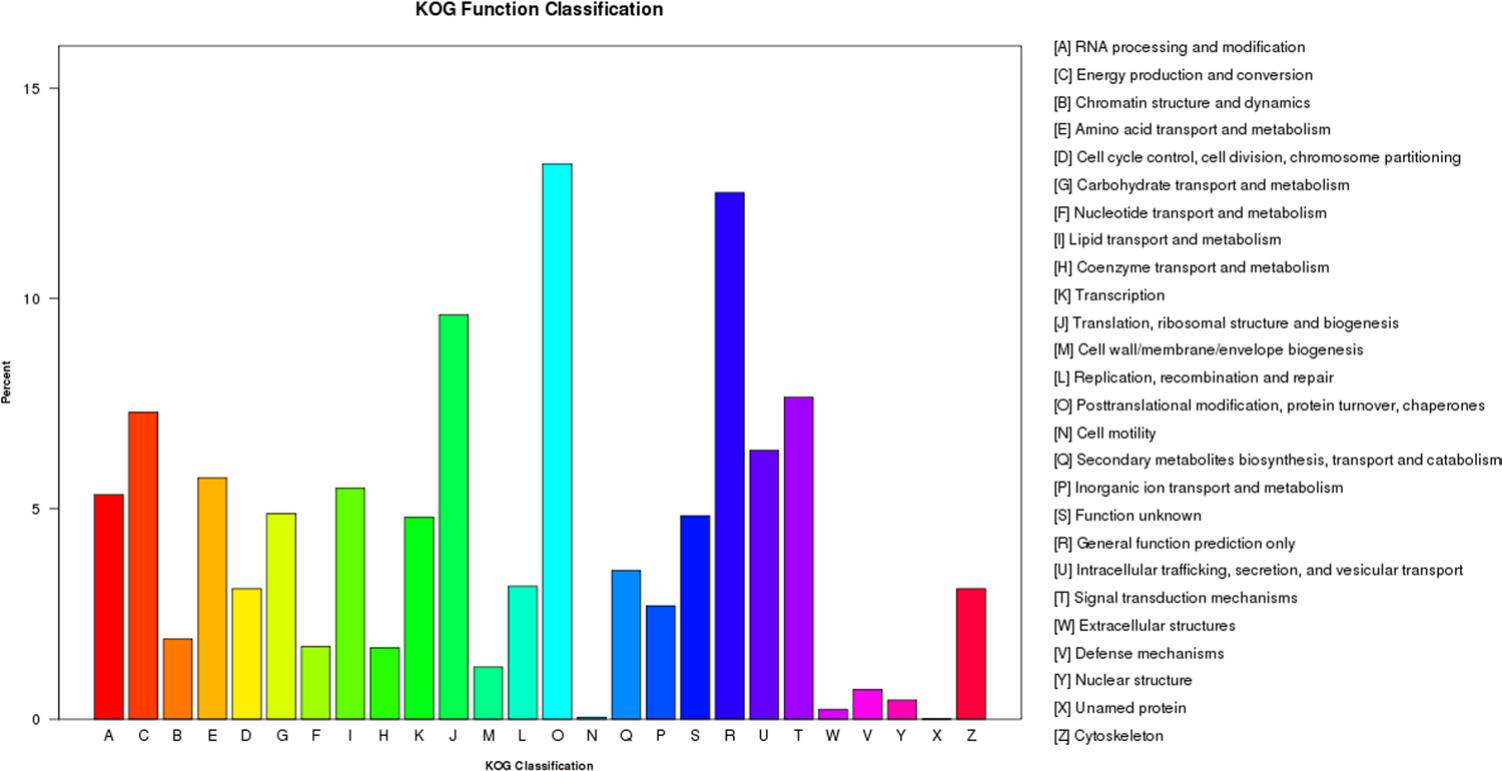
KOG annotation of putative proteins. All 11,866 putative proteins showing significant homology to those in KOG database were functionally classified into 26 molecular families. Y axis shows number of genes in a specific functional cluster.

After comparing the Unigene and KOG databases, the annotated functions of 11,866 Unigenes were predicted and classified for statistics, as shown in Fig. 2. The results showed that among the 26 KOG classifications, post-translational modification, protein turnover, and molecular chaperones were the maximum, followed by general function presumption, translation, ribosome structure and biogenesis, energy production and conversion, signal transduction mechanisms. And proteins with unnamed eukaryotic cells were the minimum (only one Unigene). This study focused on biosynthesis, transport, and decomposition of the secondary metabolism with 420 Unigenes annotated (3.54%).

### Analysis of differentially expressed genes in VPA vs VPC

**Figure 3:**
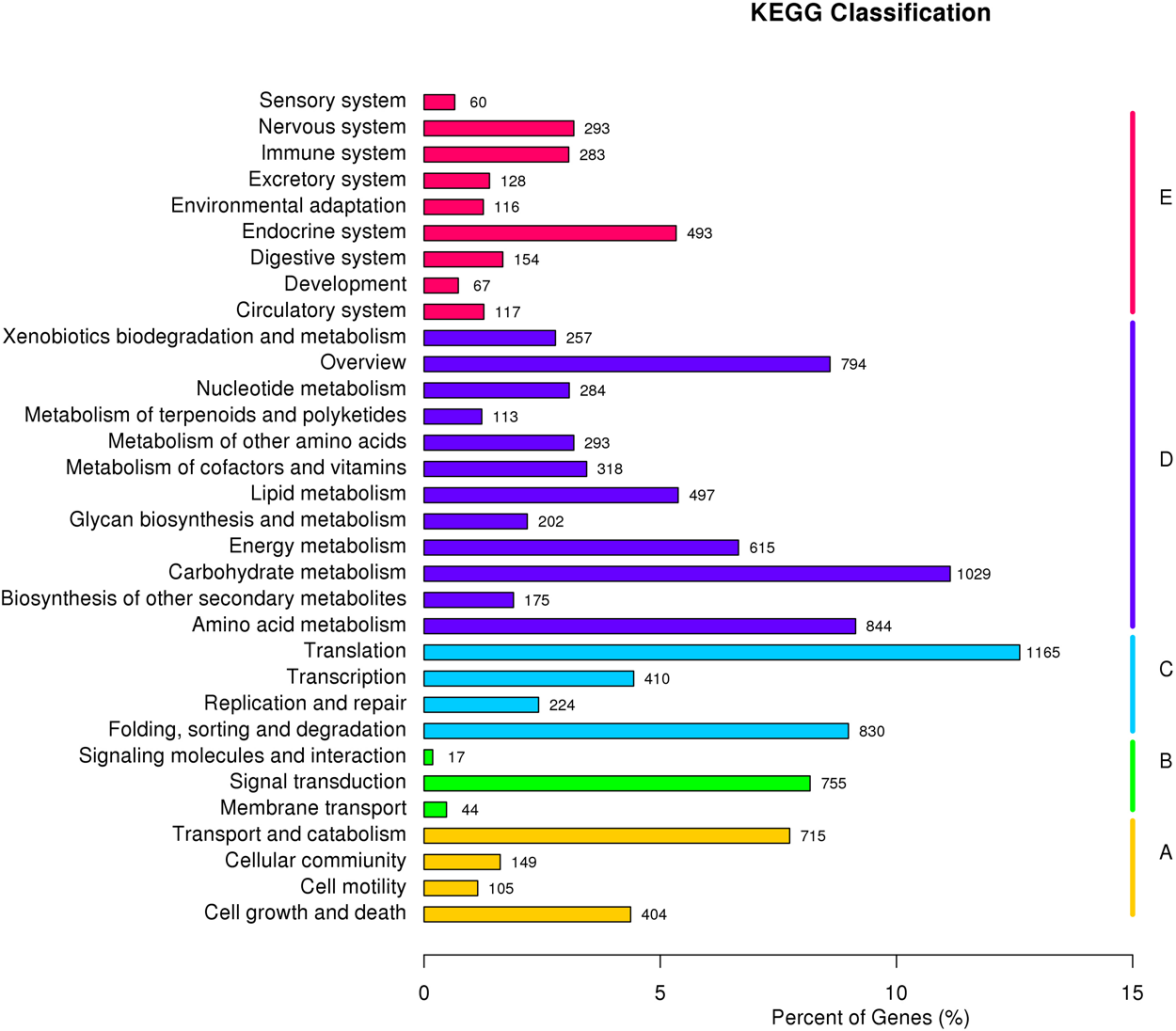
KEGG annotation of putative proteins. Distribution of genes in different KEGG categories.

**Figure 4:**
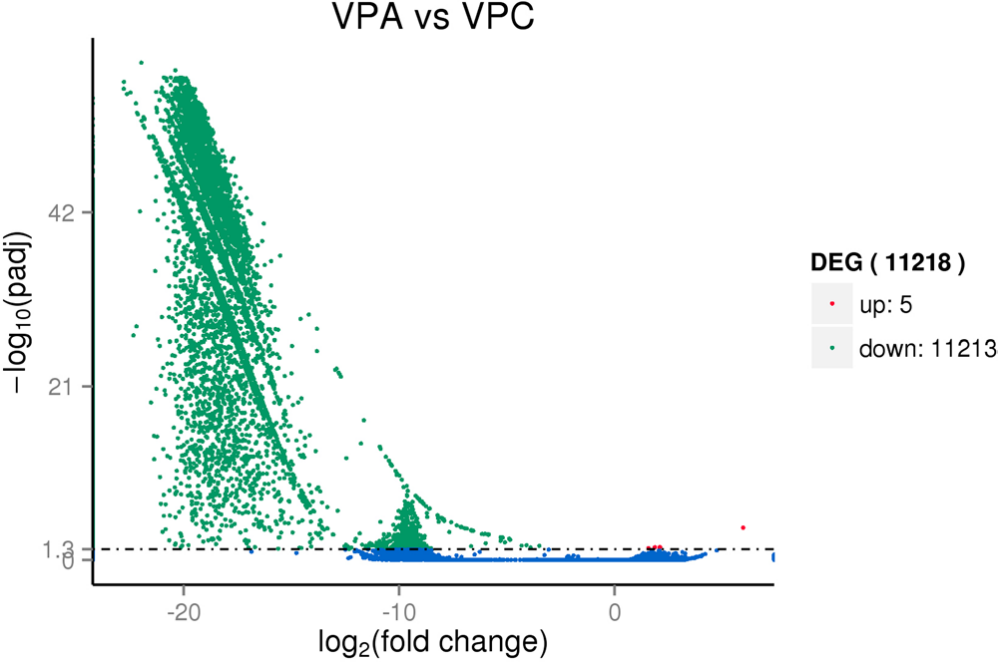
Up-regulated and down-regulated differentially expressed genes in VPA vs VPC. OW7.8 is represented by VPA and M1 is represented by VPC. Differentially expressed genes (DEGs) were determined using a threshold of FDR ≤ 0.001 and |log2Ratio| ≥ 1. Red spots represent up-regulated DEGs and green spots indicate down-regulated DEGs. Blue spots indicate genes that did not show obvious changes in expression levels between two strains.

Unigene was analyzed by kobas software for KEGG-PATHWAY enrichment of each group of difference analysis results, as shown in Fig. 3. 11,218 DEGs were obtained with OW7.8 as VPA and M1 as VPC (OW7.8 is represented by VPA and M1 is represented by VPC), of which 5 were up-regulated and 11,215 were down-regulated (Fig. 4). The up-regulated genes were associated with growth and development, while the down-regulated genes were the maximum in Glycerophospholipid metabolism, which was associated with 97 DGEs, followed by 95 DGEs in Other glycan degradation pathways and 88 DGEs in Basal transcription factors. 86 DEGs were enriched in Glycosaminoglycan degradation and 85 DGEs were enriched in Butanoate metabolism.

### Screening and cluster analysis of differentially expressed genes related to SW synthesis in *A*. *oxytropis*

**Figure 5:**
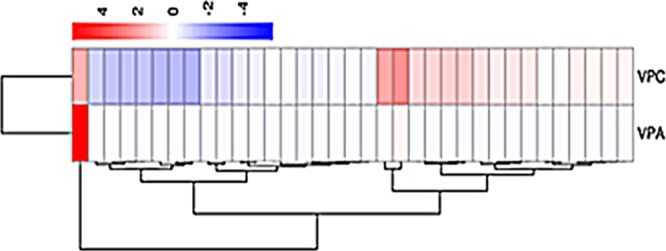
Analysis of differentially expressed Unigenes in *A*. *oxytropis*. Similarity of the expression profiles between genes with hierarchical clustering is shown in the heat map. Intensity of color indicates expression levels, and the two major clusters represent the VPA and VPC.

There are many unknown details in the SW biosynthetic pathway of the endophytic fungus. 41 DEGs involved in SW biosynthesis were screened according to RNA-Seq data. Cluster 3.0 software was used for cluster analysis of the selected DEGs. The expression patterns of each gene were shown in Fig. 5. Different colors indicated different levels of expression changes. Red indicated up-regulation of gene expression and blue indicated down-regulation of gene expression. And deeper color indicated more significant up-regulation or down-regulation of gene expression. A total of 41 DEGs related to SW synthesis were screened out based on RNA-seq. Compared with OW7.8, the expression of DEGs was down-regulated in M1.

RNA-seq analysis revealed that 7 different enzymes catalyzed the biosynthesis of SW. Saccharopine reductase played an important role in SW synthesis, and the annotation involved 5 genes. While 1-indolizidineone was the direct precursor of SW and hydroxymethylglutaryl-CoA lyase played a key role. The annotation involved three genes, mainly catalyzing the hydroxylation of 1-indolizidineone to form SW.

### The putative SW biosynthetic pathway in *A*. *oxytropis*

**Figure 6:**
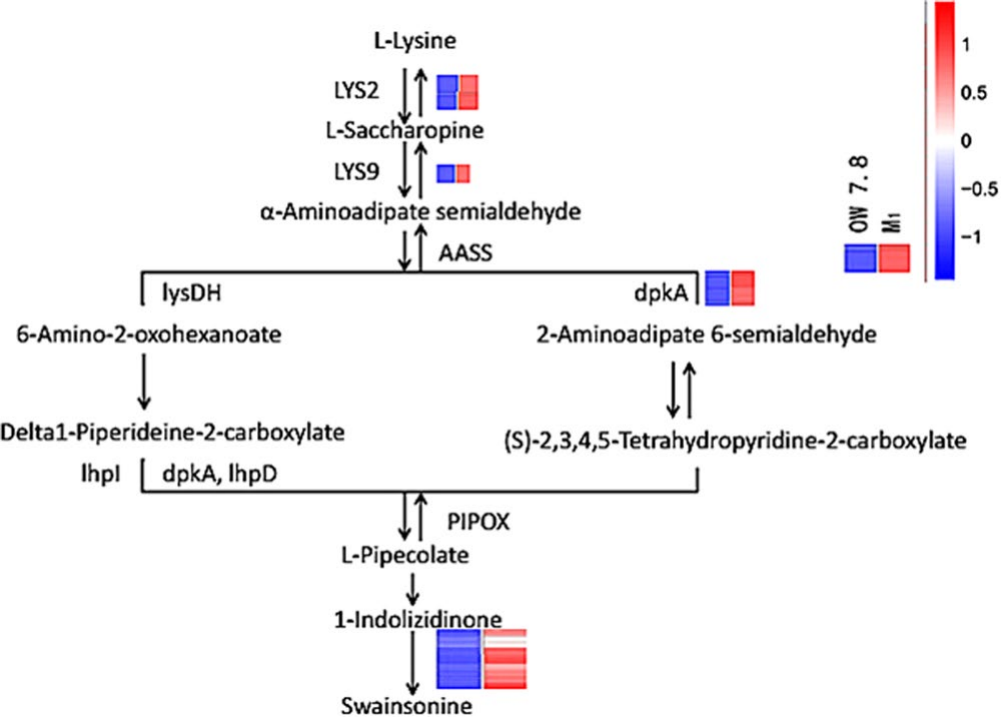
The putative swainsonine biosynthetic pathway in *A*. *oxytropis*. Enzymes involved in this pathway: LYS2/LYS9/lysDH: saccharopine dehydrogenase (NADP+, L-glutamate forming) [EC:1.5.1.10]; AASS: alpha-aminoadipic semialdehyde synthase [EC:1.5.1.8 1.5.1.9]; dpkA/lhpD:delta1-piperideine-2-carboxylate reductase [EC:1.5.1.21]; dpkA:lysine 6-dehydrogenase [EC:1.4.1.18]; lhpI: 1-piperideine-2-carboxylate/1-pyrroline-2-carboxylate reductase [NAD(P)H] [EC:1.5.1.1]; PIPOX: sarcosine oxidase/L-pipecolate oxidase [EC:1.5.3.1 1.5.3.7].

KEGG analysis of DEGs from RNA-Seq revealed that SW biosynthetic pathway was related to biosynthesis of other secondary metabolites, metabolism of cofactors and vitamins, metabolism of other amino acids, metabolism of terpenoids and polyketides and other pathways. Putative SW biosynthesis pathway of the fungus was shown in Fig. 6. Five genes related to saccharopine reductase/dehydrogenase were involved in the SW biosynthesis pathway of fungus. The two-step reaction of L-lysine → saccharopine → α-Aminoadipate semialdehyde was catalyzed by saccharopine dehydrogenase. In the P2C pathway, α-Aminoadipate semialdehyde was catalyzed by saccharopine dehydrogenase to produce 6-Amino-2-oxohexanoate, which was then converted to L-Pipecolate under the catalyzing of 1-piperidine-2-carboxylate/1-pyrroline-2-carboxylate reductase [NAD(P)H] and delta 1-piperidine-2-carboxylate reductase; and in the P6C pathway α-Aminoadipate semialdehyde, → 2-Amino-2-oxohexanoate → (S)-2,3,4,5-Tetrahydropyridine-2-carboxylate → L-Pipecolate. The synthesis of 1- ketone indolizidine by piperidine acid involved a multi-step reaction, and the details were unknown. 1-indolizidineone was a direct precursor of SW synthesis. Its hydroxylation maybe catalyzed by hydroxymethyl glutaryl-CoA lyase and other hydroxylase.

### To validate the expression patterns related to SW synthesis and correlation analysis of SW content in mycelia

**Figure 7:**
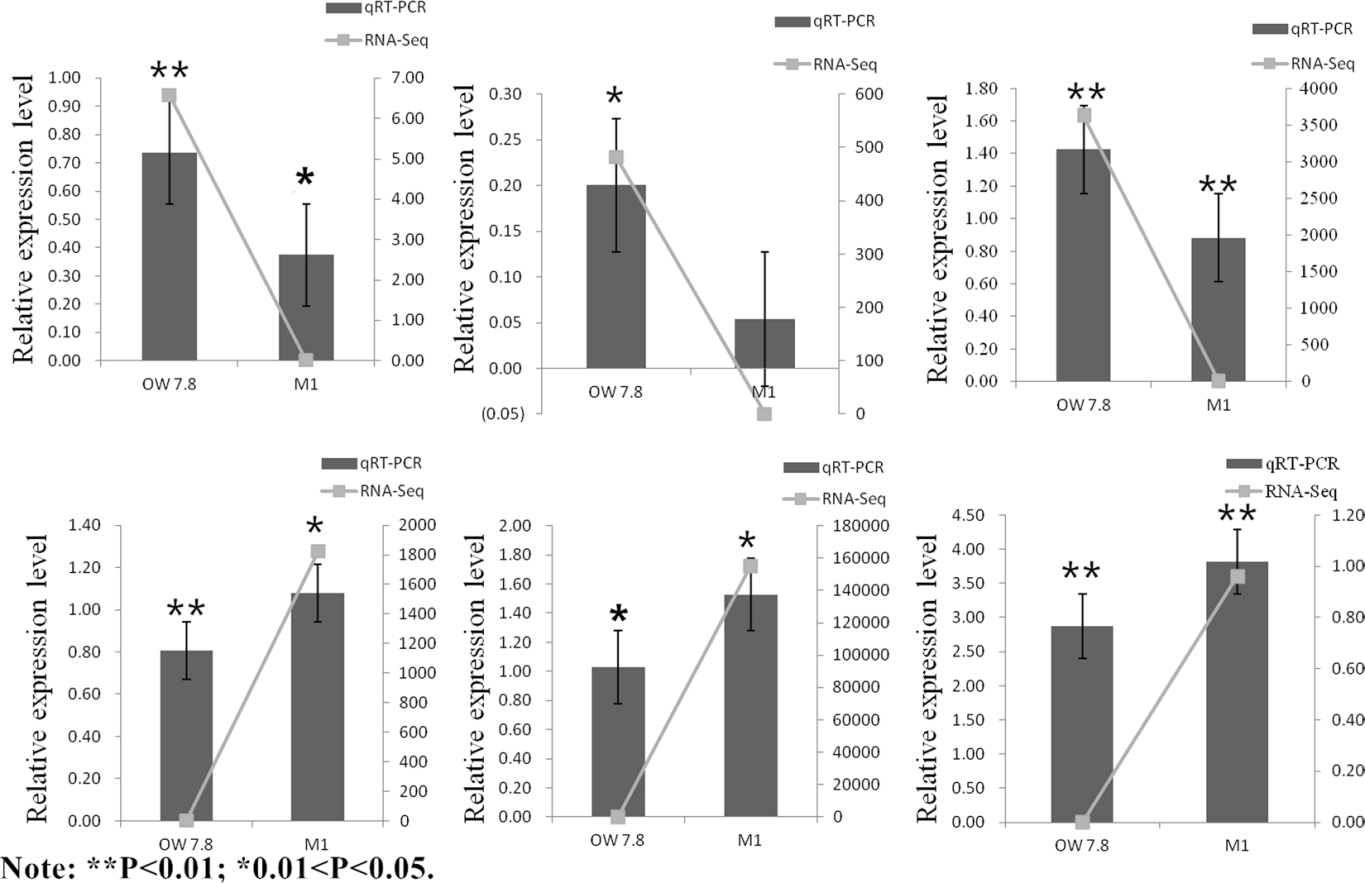
Expression profiles of six genes. The X axis represents the different genotype strains. Lines represent the FPKM value of the transcriptome result (Y axis at right). Columns and bars represent the means and standard errors of three individual samples (Y axis at left). Each experiment was performed in triplicate.

**Figure 8:**
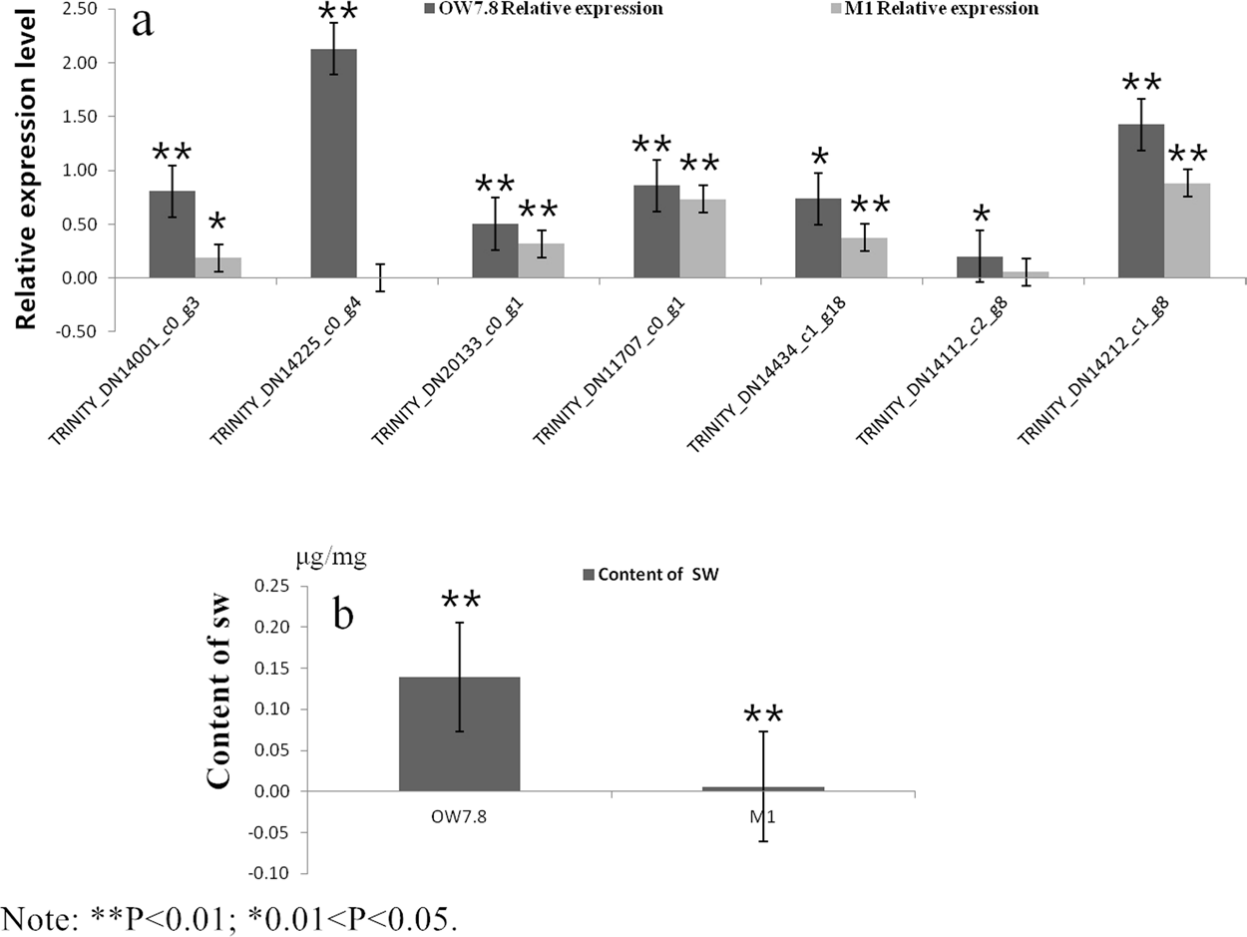
Expression profiles of SW biosynthesis-related of 7 Unigenes in *A*. *oxytropis*. (**a**) Comparison of the expression levels of seven Unigenes with SW biosynthesis. The X axis represents seven Unigenes with SW biosynthesis. Columns and bars represent the means and standard error of three individual samples (Y axis). Each experiment was performed in triplicate. (**b**) Content of SW in 20d. The X axis represents OW7.8 and M1. Lines and bars represent the means and standard errors of three individual samples (Y axis). Each experiment was performed in triplicate.

Six DEGs related to SW synthesis were randomly selected and verified by real time PCR (Fig. 7). It was found that the expression pattern of DEGs was consistent with results of the transcriptome sequencing, indicating accurate and reliable transcriptome data. In addition, all the genes selected in M1 were down-regulated, and SW levels were lower than in OW7.8. It was speculated that these genes were related to SW biosynthesis (Fig. 8).

## Discussion

Swainsonine is a toxic secondary metabolite of indolizidine produced by *A*. *oxytropis*. It inhibits the activity of alpha-mannosidase and poisons animals. It also has anti-tumor and immunomodulatory effects^1,2^. It is becoming a focus of attention. The SW content in mutant M1 was decreased by knocking out the saccharopine reductase gene in our group, indicating that saccharopine reductase plays an important role in the biosynthesis of SW. However, the biosynthetic pathway of SW in fungus is still unclear. For the first time, we sequenced OW7.8 and M1 of *A*. *Oxytropis* by Illumina HiSeq^TM^4000 high-throughput sequencing platform, which proved that both genotypes have unique expression profiles involving specific gene expression.

In SW biosynthetic pathway of fungus, saccharopine reductase^9–11^ catalyzes the transformation of lysine and saccharopine to form alpha-aminohexanedioichemialdehyde, which is one of the precursors of SW biosynthesis. So saccharopine reductase is also a key enzyme in SW biosynthesis^11^. Compared with OW7.8 strains, the saccharopine and SW decreased in M1, while lysine levels remained unchanged. The annotated genes related to SW synthesis were down-regulated in M1. We speculate that the deletion of saccharopine reductase gene hindered the synthesis of saccharopine in M1, resulting in decreased saccharopine, piperidine and 1-indolizidineone in M1, so the SW level decreased. However, as lysine is a necessary amino acid, the demand of fungus is large. In M1, lysine may be synthesized by 6-step reaction of L-Aspartate or by 11-step reaction of alpha-Ketoglutaric acid to reach the normal level so as to maintain the needs of the fungal life activities. The SW levels were increased in both OW7.8 and M1 after addition of Saccharopine, alpha-aminoadipate acid, lysine and pipecolic acid to the media^12^. In this study, 37 and 93 DEGs involved in lysine biosynthesis and degradation pathways were annotated with KEGG, respectively. In addition, 44,917 unigenes that could not be matched to the existing KEGG database were compared with the upper and lower substances in the metabolic pathways of SW and its analogs, and some of them were found to be related to SW biosynthesis. The biosynthetic pathway of tropane, piperidine, and pyridine alkaloid biosynthesis also contained SW, but the differential genes involved were not yet annotated. All of these provide evidence for the putative the SW metabolic pathway.

Based on the transcriptome sequencing data, we speculate that SW biosynthesis may have two pathways: P6C pathway and P2C pathway. The P6C pathway is L-lysine → saccharopine → α-aminoadipaldehyde → 2-aminoadipic acid-6-semialdehyde (P6C) (S) → 2-,3,4-,5-tetrahydropyridine-2-carboxylic acid, and saccharopine reductase catalyzed the first three steps. (S)-2-,3, 4-,5-tetrahydropyridine-2-carboxylic acid produced piperidine under the catalysis of pyrrolidine-5-carboxylate reductase. Then 1-oxy-indolizidine was produced to form SW under the catalysis of hydroxylase. But the specific details were unknown. The P2C pathway is L-lysine → saccharopine → α-aminoadipaldehyde → 6-aminohexanedioic acid-2-semialdehyde (P2C) → *δ*-1-hexahydropyridine-2-carboxylic acid → piperidine acid → 1-indolizidineone → SW. Saccharopine reductase also plays an important role in the pathway, which is basically consistent with the results of genome sequencing of *Undiflum oxytropis*^4,13,14^. They speculated that the enzymes involved in piperidine acid → 1-oxo-indolizidine → SW were polyketone synthase (PKS) and hydroxylase in P450 family respectively. The transcriptome data in the research suggested that the hydroxylation of 1-indolizidineone → SW may be catalyzed by hydroxymethyl glutaryl CoA lyase, while PKS and P450 are not yet enriched, and further studies are needed.

The dynamic changes of SW levels in OW7.8 and M1 as investigated previously by our group showed that SW levels in OW7.8 were always higher than those in M1^13^. In this study, SW levels of two strains cultured for 20 days were measured, and 10 DEGs were randomly selected from the genes related to SW biosynthesis for real time PCR amplification. It was found that SW levels decreased when gene expression was down-regulated, indicating that these genes were closely related to SW biosynthesis. In addition, the level of SW in this study was consistent with earlier results, while M1 was lower than OW7.8.

In the future, we are going to study the function of other enzymes related to SW biosynthesis in the endophytic fungus, compare and analyze the changes of lysine, saccharopine, piperidine acid and SW levels in order to refine the biosynthetic pathway of SW in *A*. *oxytropis*. SW-free strains may be constructed by molecular biology and gene engineering to lay the foundation for non-poisonous *Oxytropis glabra*, which is of great significance for the comprehensive management of locoweed on the grassland.

## Methods

### Sample treatment

OW7.8 and M1 are all from our laboratory. Quantitative inoculation (OW 7.8 and M1) was carried out. The 5.5 mm diameter mycelium was taken by whole puncher to be cultured on the potato sucrose agar medium. Each culture dish was added with 10 mL culture medium. Mycelium was placed in each dish. Three replicates were set up and incubated at 25 °C for 20 days. Inoculated M1 was added with hygromycin (50 mg/mL).

The total RNA of *A*. *oxytropis* was extracted by Tiangen kit for gel electrophoresis. Nanodrop was used to detect OD260/280. Qubit was used to quantify RNA and Agilent 2100 was used to detect RNA integrity. TaKaRa reverse transcription kit was used to synthesize cDNA.

### Library preparation for Transcriptome sequencing

A total amount of 1.5 µg RNA per sample was used as input material for the RNA sample preparations. Sequencing libraries were generated using NEBNext® Ultra™ RNA Library Prep Kit for Illumina following manufacturer’s recommendations and index codes were added to attribute sequences to each sample. The mRNA was enriched by magnetic beads with Oligo (dT). Then fragmentation buffer was added to break the mRNA into short fragments. The first strand of the cDNA was synthesized by random hexamers with mRNA as the template. Then the second strand was synthesized with buffer, dNTPs, DNA polymerase I and RNase H.

Second strand cDNA synthesis was subsequently performed using DNA Polymerase I and RNase H. Remaining overhangs were converted into blunt ends via exonuclease/polymerase activities. After adenylation of 3’ ends of DNA fragments, NEBNext Adaptor with hairpin loop structure were ligated to prepare for hybridization. In order to select cDNA fragments of preferentially 250~300 bp in length, the library fragments were purified with AMPure XP system. Then 3 µl USER Enzyme was used with size-selected, adaptor-ligated cDNA. Then PCR was performed with Phusion High-Fidelity DNA polymerase, Universal PCR primers and Index (X) Primer. At last, PCR products were purified and library quality was assessed on the Agilent Bioanalyzer 2100 system. When they were qualified as expected, sequencing was completed on the Illumina HiSeq^TM^4000 sequencing platform^15–29^. Then annotate the sequencing results with Nr, Nt, GO, KEGG and other public database annotations^30–32^.

The transcriptome data were analyzed to screen differentially expressed genes (DEGs), and the SW biosynthesis pathway in the fungus was speculated.

### The real-time PCR to validate the Unigenes expression patterns

Ten differentially expressed genes were randomly selected according to the cDNA sequences, and primers were designed with Primer Premier 5.0. The primers were synthesized by Sangon Biotech (Shanghai) Co., Ltd.

The PCR reaction system (25 μL): TB Green Premix Ex Tap II (TliR NaseH Plus) 12.5 μL; cDNA template 2 μL (0.1 g/L); primer 0.5 μL (10 μM); ddH_2_O 8.5 μL.

PCR amplification procedures: 95 °C, 30 s; 95 °C, 5 s, 60 °C, 0.34 s, 40 cycles; 95 °C, 15 s; 60 °C, 1 min; 95 °C, 15 s. Each sample was set up with 3 biological repetitions and 3 technical repetitions.

### Extraction and detection of SW from *A*. *oxytropis*

The OW7.8 and M1 strains were cultured for 20 days, and the SW in the mycelium was extracted and detected, and 3 replicates were set up for each sample, and SW levels were detected by HPLC-MS^3,14^. The data were analyzed by SPSS Statistic 17.0 (www.ibm.com/software/analytics/spss).
